# 

**Published:** 1890-09

**Authors:** 


### EDITORIAL.

### Personal.

Dr. W. St. George Elliott, of No. 39 Upper Brook street, London W., England, we regret to learn, will retire from the practice of dentistry, October 1st.

Dr. Elliott has for many years been one of the most prominent practitioners in London, and has, during his professional career, done much for the development and maintainance of his chosen calling. He has established for himself an enviable reputation wherever dentistry is known and practiced. The profession, even in London, can ill-afford to lose such men, for his place will not be easily filled.

He leaves London for Washington City, D. C., where he will make his future home, and enjoy to the full the fruits of his past labor.

Drs. William and J. L. Mitchell will succeed him in his London practice and will occupy the office with which Dr. Elliott has so long been identified. And we trust their success may be all that they can desire.

---

THERE will very soon, be issued from the press of the J. B. Lippencott Publishing Co., the 5th Edition, thoroughly revised with, additions a system of Oral Surgery, being a treatise on the disease and surgery of the mouth, jaws, face, teeth, and associate parts, by James E. Garretson, A.M., M.D., D.D.S., Dean of the Philadelphia Dental College.

This work will be illustrated with numerous wood cuts and steel plates. Those familiar with the former editions of this work, and with the ability of the author, will look with interest for this edition. This has been a standard work with the profession, and in all our institutions of learning for many years, and this fact makes its issue a matter of special interest to the profession throughout this country and others as well. We will have something more definite to say upon the work after its issue.

### State Board of Dental Examiners.

The annual meeting of the Ohio Board of Dental Examiners will be held, beginning at 12 o'clock on the last Tuesday in October, in Columbus.

It is to be hoped that all interested will make a note of this fact, and be promptly on hand at that time.

### Ohio State Dental Society.

The annual meeting of this body will be held in Columbus on the last Tuesday of October next. Preparations are being made for a large and interesting meeting, as it is to be hoped that every member will be sufficiently interested to use his best efforts to make the meeting eminently successful. There are many subjects that ought to receive earnest attention on that occasion.

The subject of securing an amendment to the law for regulating the practice of dentistry in this State was before the last meeting of this body for consideration. This resulted in the appointment of a committee to take the matter under advisement and take such action as in their judgment may seem necessary. The committee made some preparation as we understand, but owing to the peculiar circumstances attending the last session of the legislature it was not thought advisable to present the matter there; and action in that body was deferred to a more favorable time. The committee will be able to report progress at this meeting, and the subject will be further considered if thought necessary.

Another matter that should receive the attention of the society is the organization of auxiliary societies throughout the State. This subject ought to receive more attention than hitherto. A very small proportion of the dentists of the State are engaged or even interested in association work. Many of the very best men of the profession are still standing outside. Efforts ought to be made to enlist the co-operation of all such, and there is perhaps no better method than to secure local societies throughout the

State. There is, we think, a general waking up in the profession throughout the State in reference to the State Society and a determination on the part of some at least, that it shall be improved and made more efficient.

Twenty years ago it was a very active body, and accomplished most excellent work. It has been less active for some years past, but the indications now are that new energy will be put forth by it.

A programme will be issued very soon and sent to the profession throughout the State, and we trust invitations will be extended to the profession in our neighboring states.

### Personal—Removal.

Dr. C. W. Spalding, M.D., D.D.S., for many years a resident and practitioner of dentistry in the city of St. Louis, Mo., has removed and taken up his residence in Riverpoint, R. I. He has been gradually withdrawing from the practice of dentistry for the past three years feeling that retirement and rest would be more in accord with his feelings than the care and anxiety of an active practice, he has retired to enjoy the pleasures of a well spent professional life. Dr. Spalding was in the practice of dentistry for about fifty years; he was one of the leading practitioners in St. Louis and had a very large practice, which he conducted upon high ethical principles. Dr. Spalding never stooped to any unprofessional methods, always maintained the honor and dignity of his profession. His reputation is co-extensive with the profession in this country at least. He at one time occupied the chair of physiology in the Ohio College of Dental Surgery; he was an excellent lecturer and teacher and was master of any subject he attempted to teach. He was subsequently connected with other colleges and was equally successful in his work there. The doctor can rest assured that he will ever have an honored place in the memory and affections of his professional brethren throughout this broad land. Long may he live to enjoy the satisfaction accruing from a career full of good works and labors of love.

A NEW fabric in surgical dressing has been invented in England, the very peculiar meshes of which are said to effectually exclude micro-organisms while permitting perfect ventilation. This material is elastic, pliable, very absorbent, durable, and may be washed. It is well adapted for hygienic underwear in place of flannel.

### CONTENTS.

### COMMUNICATIONS.

### THE DENTAL REGISTER,

A MONTHLY MAGAZINE DEVOTED TO THE INTERESTS OF THE

### DENTAL PROFESSION.

*Terms Reduced to $2.00 Per Year. Single Copies, 25 Cents.*

### EDITED BY PROF. J. TAFT, D.D.S.

—AND—

### Published by SAM'L A. CROCKER & CO., Ohio Dental and Surgical Depot.

The volume commences in January and closes in December.

The REGISTER being issued monthly is always fresh, therefore Indispensable to the practitioner who desires to keep posted up with the new inventions and improvements which are daily developed. Neither expense nor effort will be spared to give value and variety to its contents. Articles will be illustrated with well executed engravings whenever they will add to the interest or assist to explanation.

Its extensive circulation and frequency of issue make it one of the BEST DENTAL ADVERTISING MEDIUMS in the United States.

All subscriptions must begin with January or July.

Local or special notices, five lines or less, will be inserted for $3 each insertion; over five lines, 50 cts. per line.

All advertising bills payable in advance, or at furthest, after the issue of the first number containing the advertisement. All transient advertisements to be paid for in advance.

☞CONTRIBUTIONS SOLICITED.

Exclusively Editorial Communications should be addressed to the Editor.

The latest number of the REGISTER may be ordered with or without other goods at any time, and all letters should be addressed to

SAM'L A. CROCKER & CO., Ohio Dental and Surgical Depot, Cincinnati, O.

